# Cd(II)-based complex loaded with drug doxorubicin hydrogels against leukemia and reinforcement learning

**DOI:** 10.1038/s41598-024-61809-6

**Published:** 2024-05-18

**Authors:** Mo Chen, Danhui Chen, Guanyu Li, Yong Wu

**Affiliations:** Fujian Provincial Key Laboratory on Hematology, Fujian Institute of Hematology, Fujian Medical University Union Hospital, Fuzhou, Fujian China

**Keywords:** Complex, Doxorubicin, Hydrogels, Reinforcement learning, Biochemistry, Chemical biology, Molecular biology, Chemistry

## Abstract

A new 3D metal–organic frameworks [Cd_6_(L)_4_(bipy)_3_(H_2_O)_2·_H_2_O] (**1**) was gained by employing Cd(II) and organic ligand [H_3_L = 4,4′,4′′-(benzene-1,3,5-triyltris(oxy))tribenzoic acid)benzene acid; bipy = 4,4′-bipyridine] in the solvothermal condition, which has been fully examined via single-X ray diffraction, FTIR and elemental analysis and so on. Using natural polysaccharides hyaluronic acid (HA) and carboxymethyl chitosan (CMCS) as raw materials, we successfully prepared HA/CMCS hydrogels and observed their internal micromorphology by scanning electron microscopy. Using doxorubicin (Dox) as a drug model, we synthesized a novel metal gel particle loaded with doxorubicin, and their encapsulation and release effects were studied using fluorescence spectroscopy, followed by further investigation of their components through thermogravimetric analysis. Based on this, the therapeutic effect on leukemia was evaluated. Finally, an enhanced learning method for automatically designing new ligand structures from host ligands was proposed. Through generative modeling and molecular docking simulations, the biological behavior of the host and predicted cadmium complexes was extensively studied.

## Introduction

Leukemia is a group of malignant clonal diseases of hematopoietic stem cells. Clonal leukemia cells proliferate and accumulate in the bone marrow and other hematopoietic tissues due to uncontrolled proliferation, impaired differentiation, impaired apoptosis and other mechanisms, and infiltrate other non-hematopoietic tissues and organs, while inhibiting normal hematopoietic function^1^. Clinically, different degrees of anemia, bleeding, infection and fever, as well as enlargement of liver, spleen, lymph nodes and bone pain can be seen. According to the degree of differentiation of leukemia and the length of natural course, leukemia can be divided into acute and chronic leukemia. Acute leukemia cell differentiation is stagnant in the early stage, with primitive and early juvenile cells predominating, and the disease develops rapidly, with a duration of several months^2^. Chronic leukemia cell differentiation is better, with naïve or mature cells as the mainstay, the development is slow, and the disease lasts for several years. According to the classification of the lesion cell series, including the myeloid system of granulocytes, monocytes, erythrocytes, megakaryoblasts and lymphoid system of T and B cell lines. Clinically, leukemia is often classified into lymphocytic leukemia, myeloid leukemia, mixed cell leukemia and so on.

In the past decades, the study of metal–organic frameworks (MOFs) has gained increasing interest because of the interesting structure features and specific functional properties: segregation, conservation, catalysis, luminescence and magnetism, and others^3–5^. It is widely recognized that the properties of MOFs are usually controlled mainly by their architecture, with extraneous factors (molar ratio of reactants, solvent system, pH, reaction thermo) being important and inherent factors (type of organic linker, configuration of ligands and properties of the central metal ion) taking a key part in governing the framework^6–10^. In terms of linker selection: polycarboxylic acid ligands are always desirable suspects for the construction of varied novel MOFs due to their multiple ligand sites and flexible ligand patterns^11–14^. In addition, based on the review of publications, aromatic N-donor linkers are often employed as auxiliary bridging, chelating and charge-balancing ligands to meet the coordination mode of metal ions used during the assembling procedure, which greatly increases the structural richness and diversity of the complexes^15–19^. The mobility and duration of the auxiliary N-ligand were also significant in tuning the coordination mode and structure^12,20,21^.

As is well known, Cd ions exhibit significant advantages in constructing MOF systems, with their unique strengths being reflected in their diverse coordination chemistry. As a transition metal, Cd can flexibly form coordination bonds with organic ligands, enabling the formation of a wide variety of complex structures in MOF construction. Additionally, cadmium forms stable complexes with some organic ligands, contributing to the preparation and stability of MOF materials. Furthermore, cadmium can provide additional functionalities in MOF construction, such as enhancing drug loading capacity and controlling the pore size and surface properties of the resulting materials. These advantages make cadmium possess unique application potential in the design and synthesis of MOFs. These distinctive characteristics make cadmium metal ions crucial components in the design and synthesis of MOFs.

To gain new functional MOFs materials, Cd(II) ions as the central metal, a symmetrical carboxylate ligand [H_3_L = 4,4′,4′′-(benzene-1,3,5-triyltris(oxy))tribenzoic acid] and a linear N-donor auxiliary linker [4,4′-bipyridine] as the organic linkers to construct the target MOFs. H_3_L has three carboxylic groups which that could offer luxuriant coordination locations and various coordination fashions, and the linear aromatic N-donor ligand (bipy = 4,4′-bipyridine) is used as auxiliary ligand act as the bridging linker to help us obtain more interesting and stable structures. Fortunately, a MOF [Cd_3_(L)_4_(bipy)_3_(H_2_O)_3_] (**1**) with 3D net framework has been successfully obtained, which has been fully characterized. **1** crystallizes in the triclinic crystal system with *P-*1 space group. Topological result shows that the whole framework of **1** could be considered as a fivnodal (4,5,6,6,6-c) 3D net with a point symbol of {3·4^6^·5^2^·6}{3·4^6^·5^3^·6^3^·7^2^}{3^2^·4^3^·5^4^·6^6^}{3^2^·4^6^·5^4^·6^2^·7}{4^3^·9^3^}.

Doxorubicin belongs to the anthracycline family of antibiotics with strong cytotoxicity, and is a first-line cancer chemotherapy drug used to treat leukemia, lung, breast, cervical, ovarian, prostate, and bladder cancers^22,23^. After local administration of doxorubicin, its utilization rate is often low due to the rapid metabolism and excretion of the body, and may be accompanied by large toxic side effects^24^. In order to improve the efficiency of drug action and reduce its potential adverse effects, the development of drug carriers is particularly important. Natural polysaccharide hydrogels have become ideal drug carriers because of their excellent biocompatibility, biodegradability and high specific surface area^25,26^. This hydrogel is not only able to effectively load the drug, but also can control the drug release rate through its unique structure, thus achieving the therapeutic goal with a smaller drug dose^27,28^. Therefore, natural polysaccharide hydrogels as drug carriers have received extensive attention and research in the field of medicine.

In this study, we designed metallic gel particles incorporating doxorubicin to assess their effectiveness against leukemia. Leukemia cells were exposed to increasing concentrations of the system, resulting in a significant upregulation of the pro-apoptotic gene Bax and subsequent induction of apoptosis.

Additionally, we explored the therapeutic potential of novel compounds with unique configurations in leukemia treatment. However, identifying specific drug molecules for targeted diseases poses challenges due to structural similarities among candidates and resource allocation uncertainties. To address this, computational models have been developed for automated discovery of new drug structures and activities. To better understand the anti-leukemia properties of our newly designed drug molecule and predicted ligands, we utilized reinforcement learning and molecular docking simulations. This comprehensive approach illuminates the efficacy of our innovative drug molecule and the potential of predicted ligands in leukemia treatment.

## Experimental section

### Materials and instruments

Any beginning material and the reagents were acquired from the market and were not purified anymore in the experiment. elemental assays for C, H and N were tested with a Perkin-Elemer 2400C elemental analyzer. Infrared spectra were measured on a Bruker EQUINOX-55 FT-IR spectrometer with KBr particles in the region of 4000–400 cm^−1^.

### Synthesis of [Cd_6_(L)_4_(bipy)_3_(H_2_O)_2·_H_2_O](1)

A mixture of 0.0342 g (0.15 mmol) CdCl_2_·2H_2_O, 0.048 g (0.1 mmol) H_3_L, and 0.015 g (0.1 mmol) bipy was prepared. The resulting mixture was heated in a 25 mL stainless steel vessel lined with polytetrafluoroethylene at 120 °C for 72 h. Subsequently, the reactor was cooled to ambient temperature to obtain colorless block-shaped crystals. The yield was 45% (based on H_3_L). Elemental analysis for **1**: anal. Calcd for C_138_ H_90_ Cd_6_ N_6_ O_39_ (%): C, 52.94; H, 2.90; N, 2.68. Found: C, 53.10; H, 2.95; N, 2.67. IR (KBr, cm^−1^): 3431 (bs), 1643 (s), 1623(s), 1590(s), 1583(s), 1478(m), 1467 (m), 1482 (s), 1328 (w), 787 (m), 684 (w), 534 (w) cm^−1^.

### Crystallographic data collection and refinement

The crystallographic dates of **1** were documented with a Bruker SMART APEX II CCD detector with graphite monochromatic Mo-Kα radiation (λ = 0.71073 Å) employing the j/ω scanning technology. The crystal architecture of **1** was anisotropically refinement on F2 with SADABS correction for absorption using SHELXTL and Olex2 programs^29,30^. The thermodynamic parameter of anisotropy is employed for non-hydrogen atoms. All the hydrogen atoms of the organoligands were computed and loaded at the desired locations. Additional particulars of the crystallographic figures are presented in Table 1.

**Table 1:** Crystallographic information and structural modifications of **1**.

Empirical formula	C_138_H_90_Cd_6_N_6_O_39_
Formula weight	3130.55
Crystal system	triclinic
Space group	*P-1*
* a* [Å]	9.9748(3)
* b* [Å]	18.5970(7)
* c* [Å]	19.2944(7)
α [°]	112.738(3)
β [°]	96.796(3)
γ [°]	98.931(3)
* V* [Å^3^]	3197.9(2)
Z	1
D_calcd._ [g cm^−3^]	1.626
μ [mm^-1^]	1.067
* F* [000]	1560
* θ* [°]	3.104–29.074
Reflections collected	31,062/14,779
GOF on *F*^*2*^	1.028
* R*_*1*_^*a*^[*I* > *2σ(I)*]	R_1_ = 0.0391, ωR_2_ = 0.0748
* WR*_*2*_^*b*^[*I* > *2σ(I)*]	R_1_ = 0.0634, ωR_2_ = 0.0842
CCDC number	2211848

(a) *R*_1_ = Σ||*F*o|–|*F*c|/Σ||*F*o|. (b) *wR*_2_ = {[Σ*w*(*F*o^2^–*F*c^2^)^2^/Σ*w*(*F*o^2^)^2^]}^1/2^.

### Preparation and characterization for doxorubicin-loaded metal gel

First, we dissolved the HA powder and CMCS powder (provided by Sinopod Chemical Reagents Co., LTD.) in 50 ml of deionized water to form a 1wt% HA solution and 3wt% CMCS solution, respectively. Then, in order to activate the carboxyl group in the HA solution, we gradually added EDC/NHS solution to the HA solution. Next, we mixed the HA mixture with 3wt% CMCS solution in equal volume ratio and stirred quickly. Subsequently, the mixture was poured into the mold and left overnight to allow the solution to fully react after molding, and it was removed from the mold to obtain the HA/CMCS hydrogel. To prepare the doxorubicin-loaded metal gel, we first soaked the Cd coordination polymer in 10 mg/ml doxorubicin solution and treated it ultrasonic for 30 min to achieve adsorption. Then, the polymer was transferred to the CMCS solution to obtain a metal gel.

After freeze-drying, the samples were sprayed with gold and the internal microstructure of the samples was observed by scanning electron microscope.

### Realtime PCR

Leukemia K562 cells were cultured in RPMI 1640 culture medium containing 10% fetal bovine serum. K562 cells were inoculated in 24-well plates at a density of 1 × 10^5^/well. After 4 h of incubation, the cells were treated with doxorubicin drug loaded with 0, 10, 20 and 50 mM of metallic gel particles containing doxorubicin drug for 24 h. The total RNA was extracted using Trizol reagent (ThermoFisher, USA). The cDNA was synthesized by EasyScript First-Strand cDNA Synthesis SuperMix (TransGen, China). The Realtime PCR was performed on CFX 96 Realtime PCR system (Bio-Rad, USA) using Luna Universal qPCR Master Mix (NEB, USA) according to manufacturer instructions.

### The generative automated model

The establishment of reinforcement learning model has been constructed using the molecule deep Q-networks (MolDQN) algorithm^31^. The features that are rewarded as reinforcement learning are the bonding ability among the drug molecules and the probe proteins, which estimates the biological activity of the drug molecule, the synthetic accessibility (SA) score, that presents the ability to be synthesized and the quantified estimation of drug similarity (QED) score, which qualifies the resistance of the drug molecule to the database of known structures. During the reinforcement learning simulation, the QuickVina 2.1 is used for predicting the binding energy, the GYM-molecule code is used for estimating the SA score (https://github.com/bowenliu16/rl_graph_generation/tree/master/gym-molecule), and the QED score is calculated by the RDKit package (https://www.rdkit.org). More details about the simulation can be found in the original paper of MolDQN^31^.

### Molecular docking

To probe the anti-leukemia activity of the newly designed Cd complex and the predicted drug molecules, the Estrogen-related Receptor-3 (ERR-Gamma) has been used as the probe protein for providing the docking pocket, where the central coordinates are 70.316, 1.159, 151.101 Å, the PDB ID is 5YSO^32^. Number of cubic grid boxes was 60. Preparation of molecule pairing was done by AutoDockTools 1.5.7 and simulation of molecule pairing was done by AutoDock 4.2. The binding pose was assessed by Lamarckian genetic algorithm (LGA), where the Cd complex was set to be semi-flexible and the ERR-Gamma receptor was made rigid.

## Results and discussion

### Structural characterization

**Figure 1:**
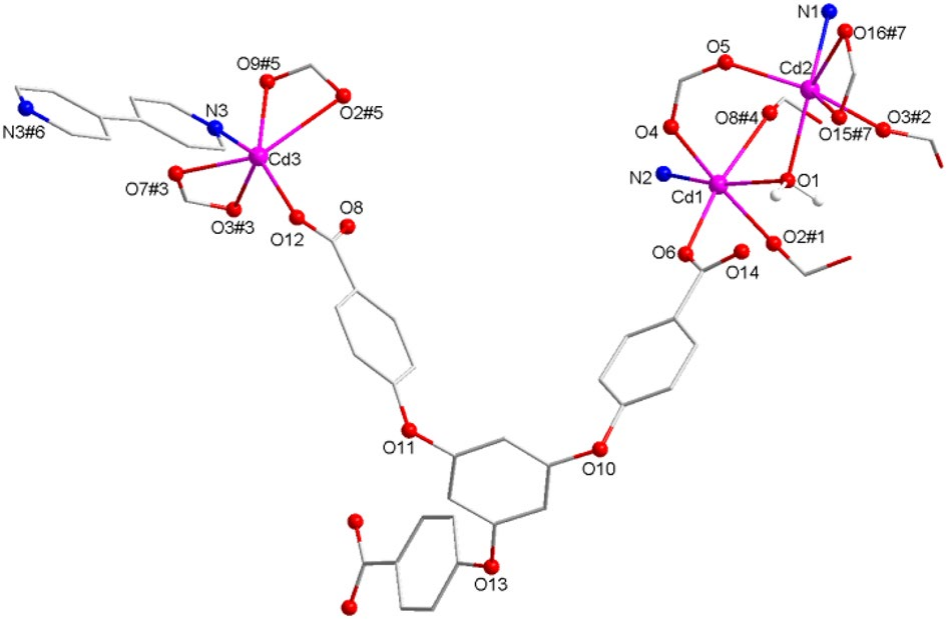
The coordinating environment of Cd(II) ions in **1**. Symmetry codes, #1: x, y, 1 + z; #2: 1−x, 2−y, 1vz; #3: x, −1 + y, z; #4: 1−x, 1−y, 1−z; #5: 1−x, 1−y, −z; #6: −1−x, −y, −z; #7: x, 1 + y, z.

The XRD data revealed that complex **1** crystallized in a trigonal crystal series with a *P-1* space group and displayed a three-dimensional dense packing structure. The unsymmetric element comprised six individual Cd(II) ions, four L3-ligands, three auxiliary bipy ligands, two coordinated water moieties, and one lattice water moiety. As depicted in Fig. 1, all Cd atoms exhibited a lusterless octahedral geometry: Cd1 atom was bound to four carboxyl O atoms of four L3 ligands, an O atom of a water molecule, and an N atom of an independent biradical ligand; Cd2 coordinated with four carboxyl O from three L3 ligands, an O from a water moiety, and an N from a biradical ligand; while Cd3 atom attached to five carboxyl O from three L3 ligands and an N from a biradical ligand. The Cd-O distance ranged from 2.148(2) to 2.605(2) Å, and the O-Cd-O angle ranged from 53.30(10)° to 176.68(9)°.

**Figure 2:**
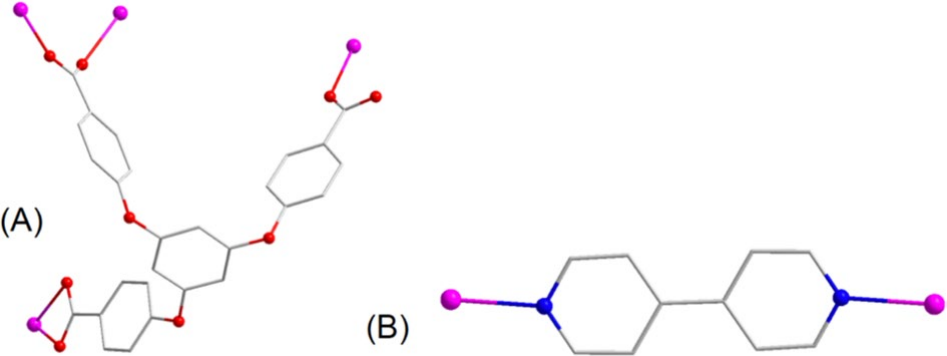
(**A**) The coordinating structure of L^3–^ in **1**. (**B**) The coordinating structure of bipy ligand in **1**.

**Figure 3:**
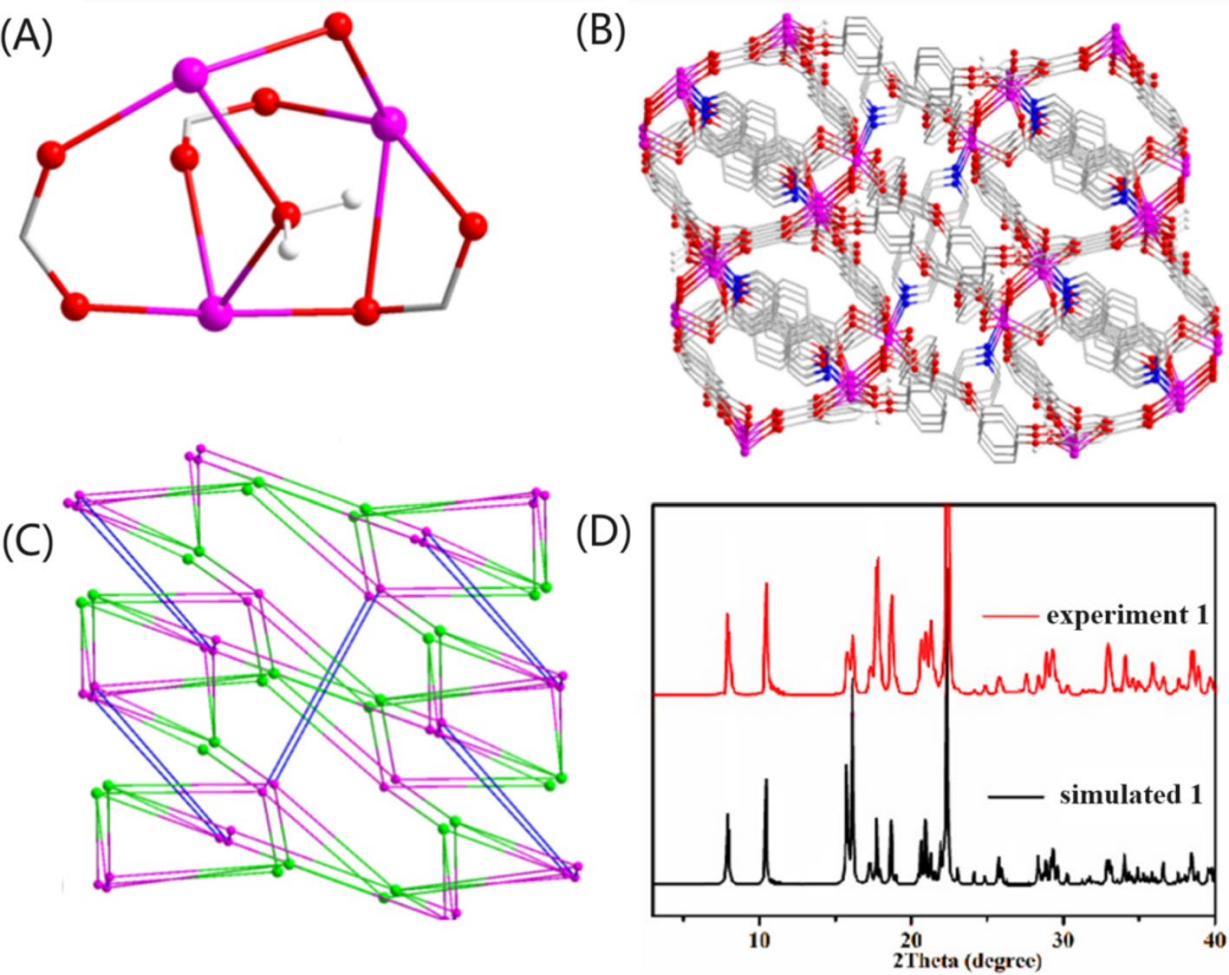
(**A**) The trinuclear cluster in **1**; (**B**) 3D architecture of **1** observed down the b-axis; (**C**) topological network of **1**; (**D**) the simulated and experiment PXRD patterns of **1**.

In **1**, the L3-ligand connects Cd(II) ions through three carboxylate groups, which adopt bidentate chelate, bidentate and monodentate bridging coordination modes (Fig. 2). Based on the rich coordination pattern, a trinuclear cluster composed of three Cd(II) ions and three carboxylate groups of the three L3-ligands has been formed (Fig. 3A). The trinuclear metal clusters are connected by L^3-^ and bipy, giving a 3D framework structure (Fig. 3B). In addition, topological analysis show that the whole structure of **1** could be considered as a five nodal (4,5,6,6,6-c) 3D topological net that has point symbol of {3·4^6^·5^2^·6}{3·4^6^·5^3^·6^3^·7^2^}{3^2^·4^3^·5^4^·6^6^}{3^2^·4^6^·5^4^·6^2^·7}{4^3^·9^3^} (Fig. 3C).

To validate the successful synthesis of the product and demonstrate the phase purity of the product, we conducted PXRD testing (see Fig. 3D). The peak positions in the PXRD patterns from both simulation and experiment exhibited excellent agreement, imparity of intensity can possibly result from prior orientation of the crystal samples, this indicated the successful synthesis of the target sample, and the synthesized sample showed good purity.

### Micromorphology of the hydrogels

**Figure 4:**
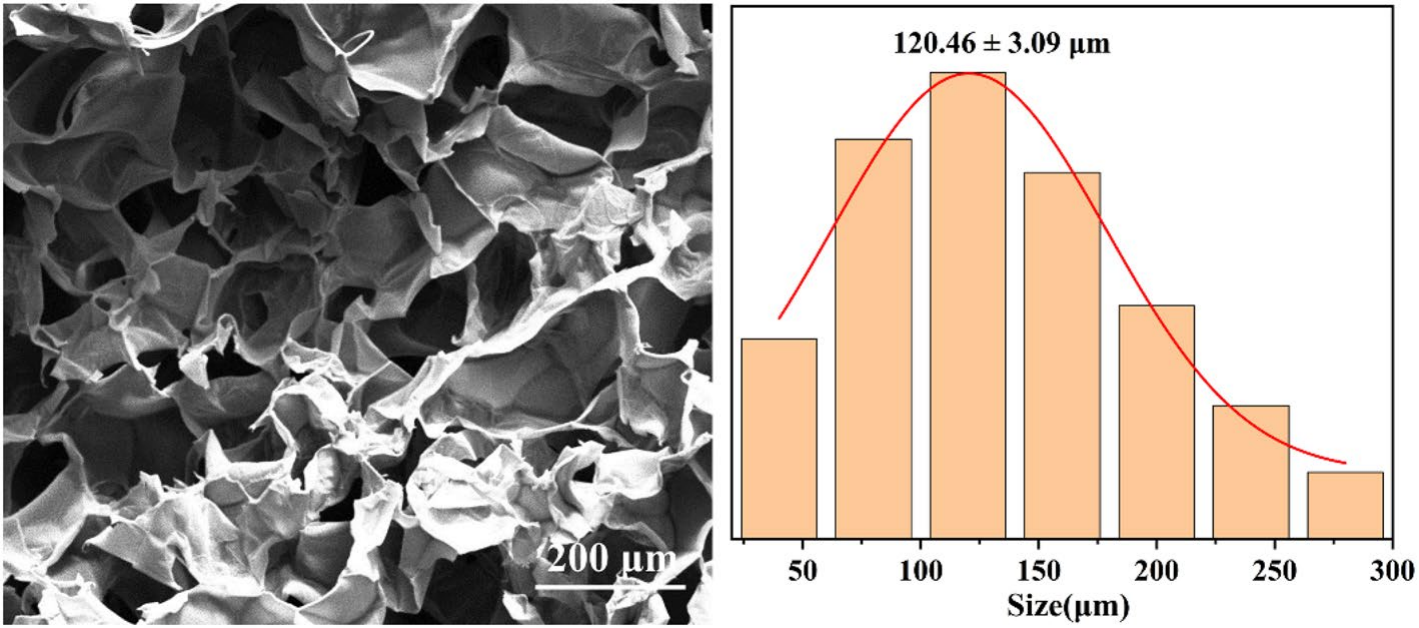
Microscopic morphology and pore size distribution of HA/CMCS hydrogels.

Hydrogels are formed by physical or chemical crosslinking of hydrophilic polymers and have excellent water absorption and water retention. This property makes hydrogels widely used in many fields, such as the manufacture of medical dressings, the preparation of lubricants and the development of drug controlled release systems. The hydrogel contains a large number of tiny interconnected pores, which not only provide sufficient loading space for the drug, but also ensure the smooth circulation of the drug during the release process. Based on the chemical synthesis method, we successfully prepared HA/CMCS hydrogels and observed their internal micromorphology by scanning electron microscopy. As shown in Fig. 4, the lyophilized HA/CMCS gel showed a clear three-dimensional porous structure. These holes were highly interconnected, forming a complex network that provides an ideal pathway for drug loading and release. In addition, we found that the pore sizes of these pores were mainly concentrated in the range of 120.46 ± 3.09 μm, which allowed the hydrogel to load and release drug molecules more efficiently.

### In vitro drug release studies

**Figure 5:**
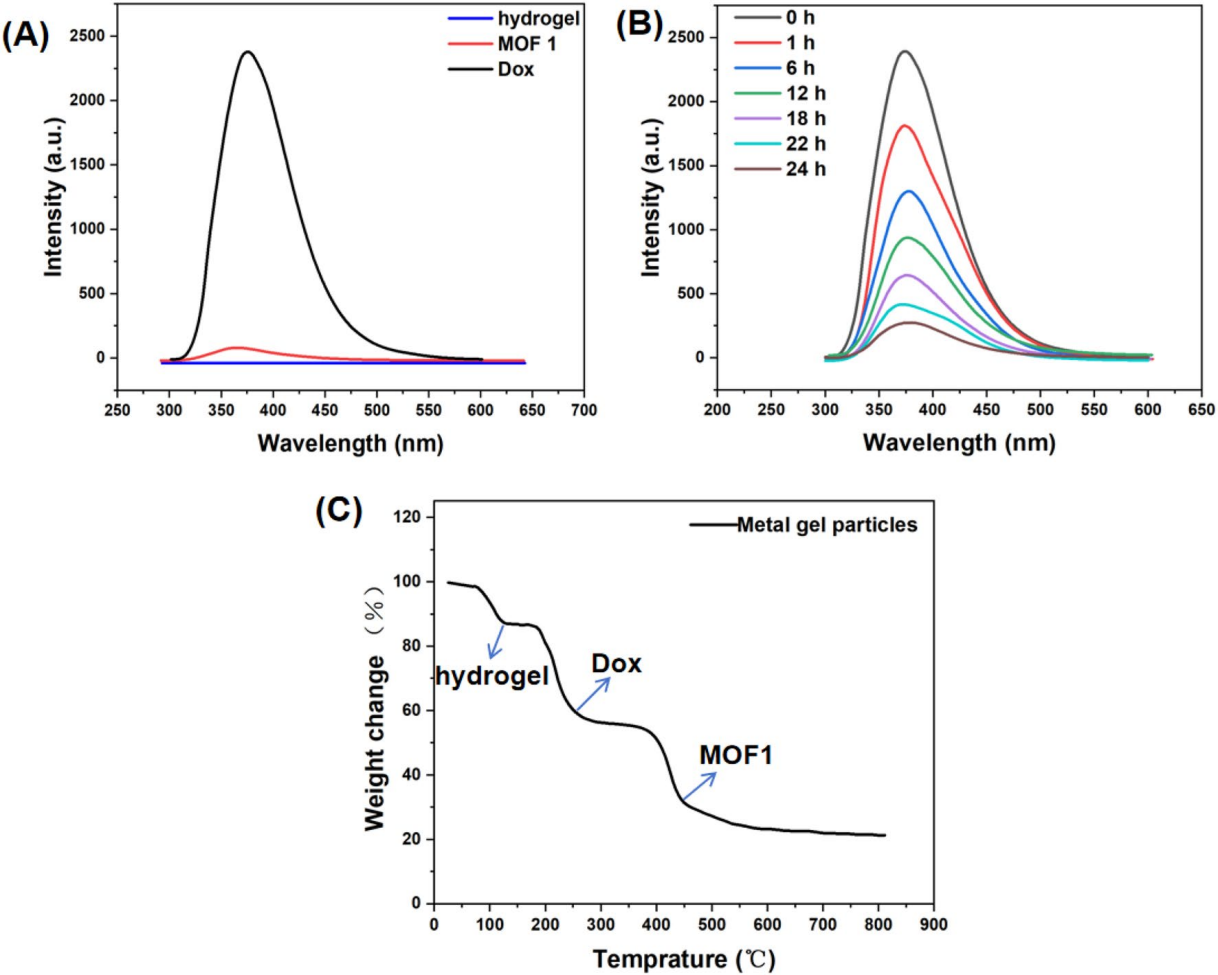
(**A**) Fluorescence intensity of different components in metal gel particles; (**B**) variation in fluorescence intensity over time during the release of Dox at the same concentration; (**C**) TGA analysis of metal gel particles.

To further verify whether the prepared metal gel particles can be effectively released in aqueous solution, we conducted fluorescence tests on hydrogel, MOF1, and Dox, respectively. The fluorescence images of each are depicted in Fig. 5A. From the images, it can be observed that Dox exhibits a typical fluorescence absorption peak at 375 nm, consistent with the literature, while both the hydrogel and MOF1 show no fluorescence response. Based on these observations, we encapsulated Dox-loaded MOF1 within the hydrogel, resulting in the appearance of a fluorescence characteristic peak at 375nm, thereby demonstrating effective encapsulation (Fig. 5B). Subsequently, we employed these novel metal gel particle drugs for related research in leukemia treatment. A typical experimental procedure is as follows: after treating leukemia cells with the same drug concentration for 24 h, metal gel particles were extracted for fluorescence testing, as shown in Fig. 5B. The fluorescence of the metal gel particles began to diminish after one hour, halved at 6 h, reduced to two-thirds at 18 h, and almost disappeared at 24 h. This indicates successful release of Dox over time, resulting in diminishing fluorescence of the metal gel particles themselves.

Additionally, we conducted thermogravimetric analysis (TGA) to further analyze the components of metal gel particles. TGA experiments were conducted under a N_2_ atmosphere in the temperature range of 20–900 °C, with a heating rate of 10 °C per minute. As shown in Fig. 5C, the initial weight loss process occurred at approximately 87 °C, corresponding to the release of the hydrogel. Subsequently, upon further heating to above 205 °C, Dox underwent decomposition. When the temperature increased to above 440 °C, further weight loss was attributed to the release of the MOF ligand.

### Metal gel particles loaded with doxorubicin significantly up-regulated the expression of Bax thereby inducing apoptosis in leukocytes

**Figure 6:**
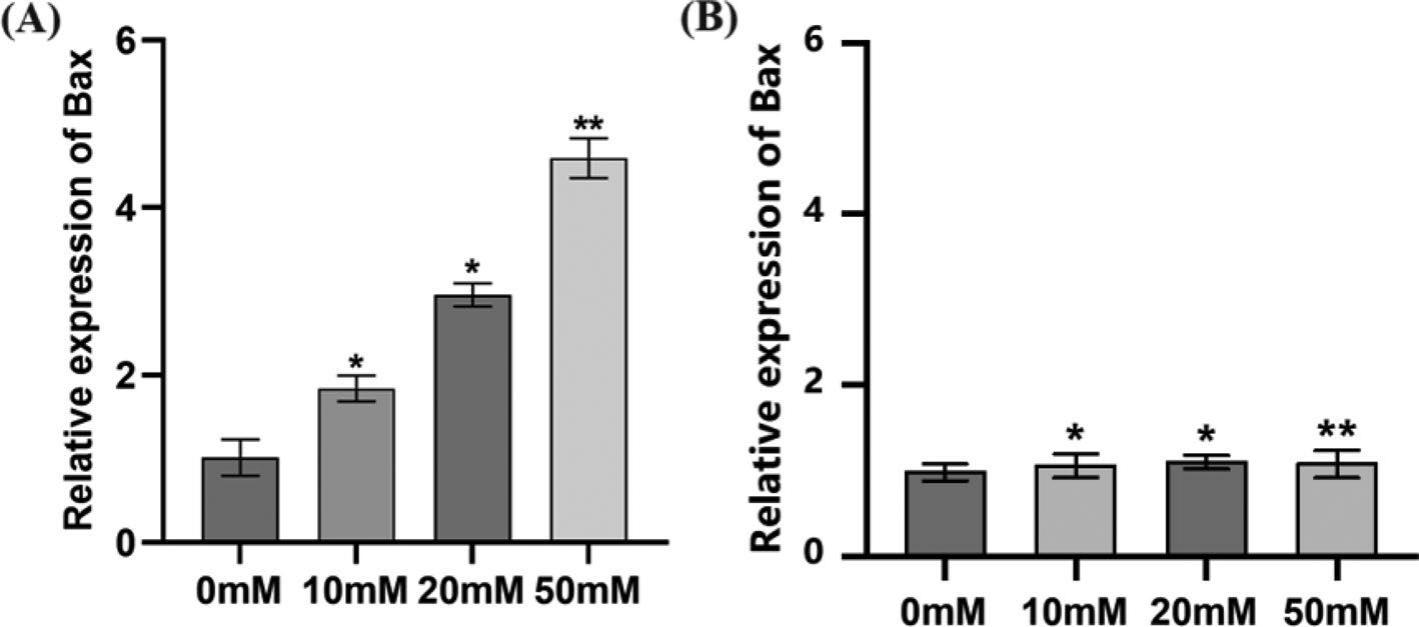
The effect of metal gel particles loaded with (**A**) and not loaded with (**B**) doxorubicin on mRNA level of Bax was determined by Realtime PCR. *Indicated P < 0.05, and ** indicated P < 0.01.

To verify whether doxorubicin-loaded metal gel particles could treat leukemia, RNA from leukemia cells K562 cells was extracted after treating the cells with different concentrations of the drug for 24 h. The expression level of the pro-apoptotic gene Bax was detected using Realtime PCR. As shown in Fig. 6A, the mRNA level of Bax was significantly increased after the cells were treated with drugs, and the change trend was drug concentration-dependent. Our results suggest that doxorubicin-loaded metal gel particles can significantly up-regulate the expression of Bax and thus induce apoptosis in leukodystrophy cells. Furthermore, to verify the therapeutic effect of unloaded drug-metal gel particles on leukemia, we treated leukemia cells with unloaded drug-metal gel particles at different concentrations under the same experimental conditions for 24 h, followed by extraction of RNA from K562 cells (Fig. 6B). Real-time PCR was used to detect the expression levels of the pro-apoptotic gene Bax. The results showed that the mRNA levels of Bax in cells remained unchanged after treatment with unloaded drug-metal gel particles, and no correlation with the metal gel was observed. This indicates that unloaded drug-metal gel particles have no effect on Bax expression and cannot induce apoptosis in leukemia cells.

### Automated ligand predictions

As has been mentioned in the methods section, three chemical properties for each predicted molecule are computed, including the binding energy, synthetic accessibility (SA) and measures of drug-likeness (QED). The successful implementation of the generative model should expect that the binding energy decreases with the number of predictions, and the SA and QED scores should increase with increase in the number of predictions. The desired structure should have low binding energy, meanwhile, has high SA and QED scores.

**Figure 7:**
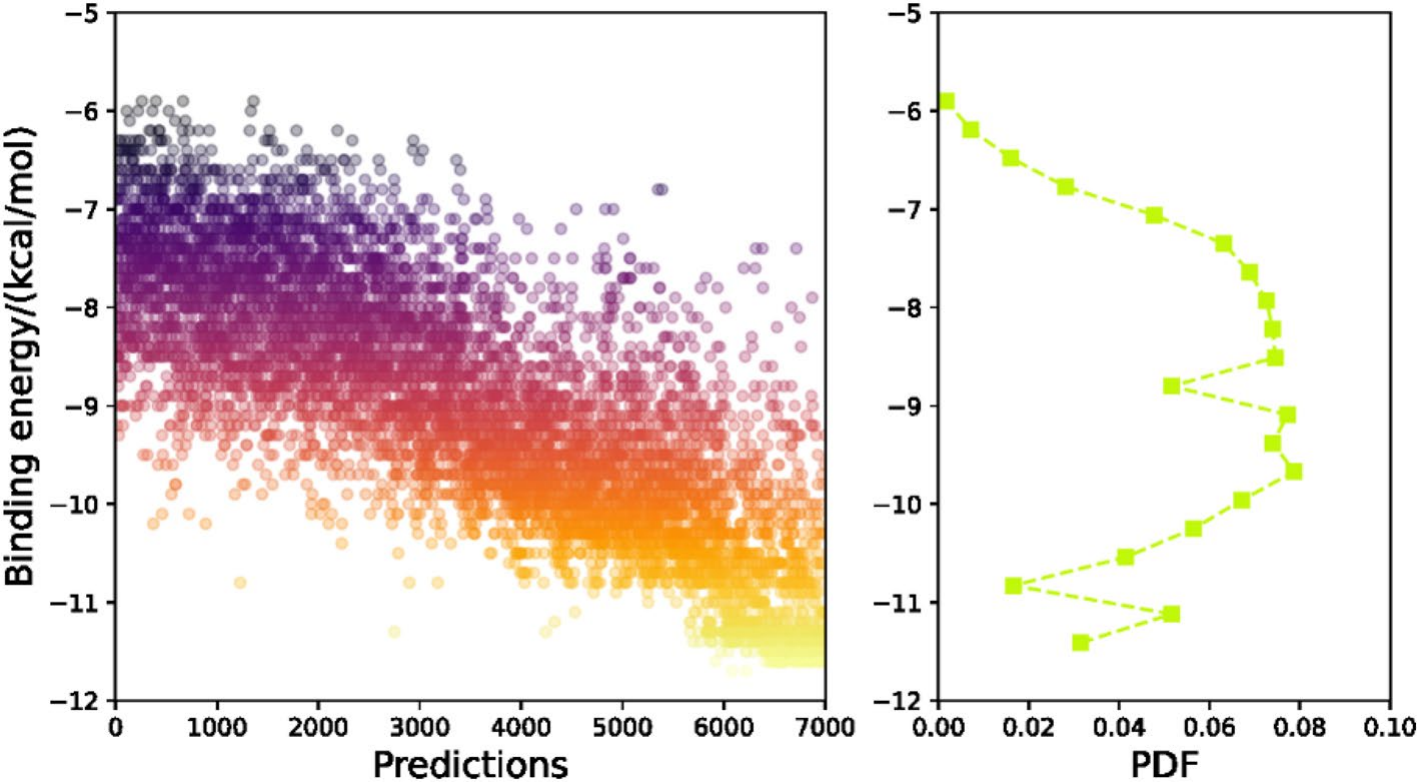
Predicted binding energies and probability distributions between ligand structures and estrogen-related receptor-3 (ERR-Gamma) (PDF).

The binding energy and the corresponding probability distribution are display in Fig. 7. From the result we can see that the binding energy range is from − 6 to − 12 kcal mol^−1^, and it monotonically decreases with the number of predictions. A broad peak is seen between − 7.5 and − 10 kcal mol^−1^. The binding energy of the host molecule is − 8.5 kcal mol^−1^, which indicates there is a lot of generated structures could not have better bioactivity toward probe protein. It can also be seen that there is a small peak around − 11 kcal mol^−1^. Thus, we choose − 10.3 kcal mol^−1^ as the benchmark for the following selection of the succeed predictions, on one hand, such a criterion enables the predicted molecule has lower binding energy than that of the host molecule. On the other hand, such a criterion guarantees about 23.0% potential candidates.

**Figure 8:**
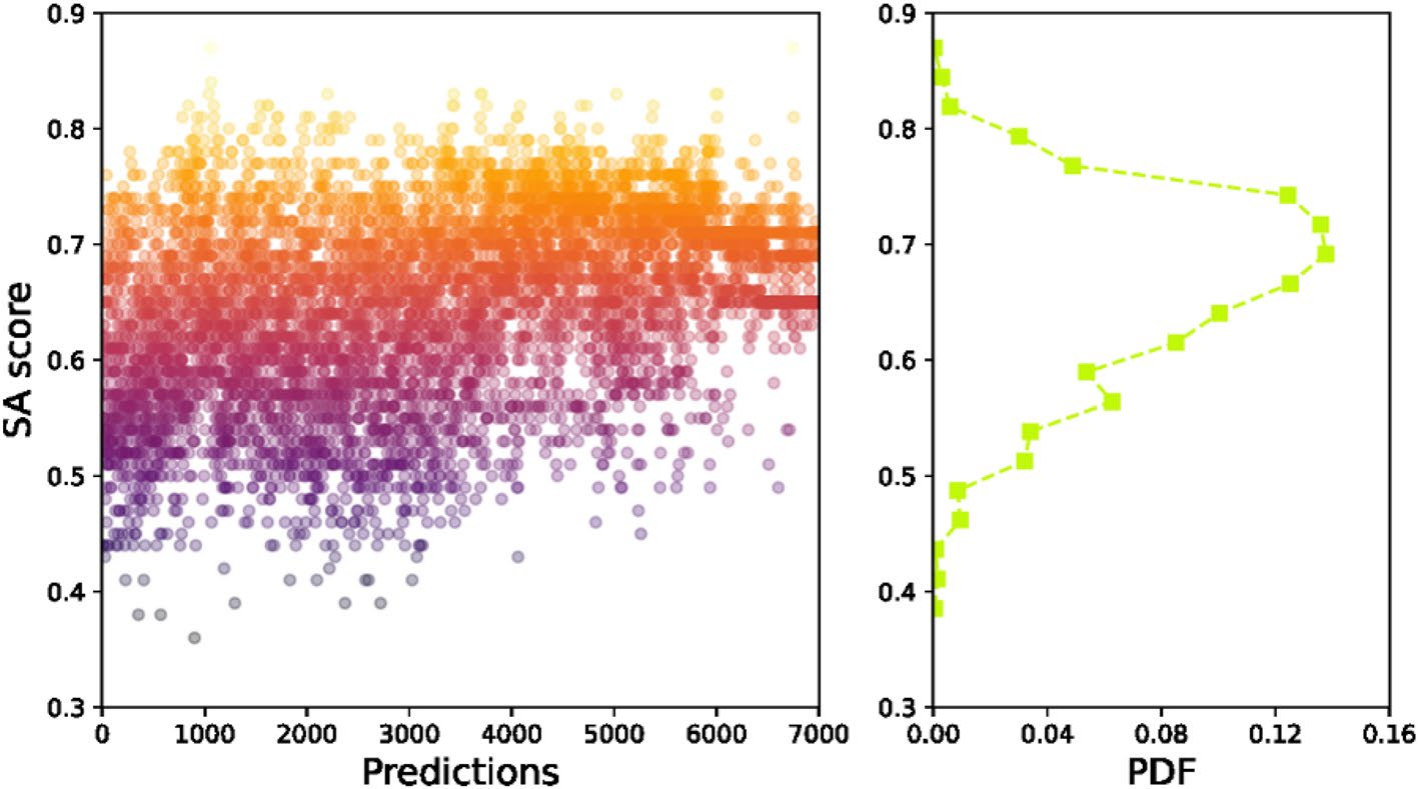
The synthetic accessibility (SA) score of the predicted ligand and the probability distribution (PDF).

In Fig. 8 the estimation of SA score for 7000 predictions and its probability distribution are shown. Different from the results that have been observed for the binding energy, the estimated SA scores aren’t presenting a clear trend, but a slightly increased trend can still be seen. The distribution of SA scored are ranging from 0.35 to 0.85, and a strong peak can be seen for SA at 0.7. As a comparison, the SA score for host molecule is 0.91, which suggests that none of the 7000 predictions cannot have a SA score as high as the host molecule. According to the probability distribution, we consider that a successful prediction should have a SA score not smaller than 0.72, and about 24.8% of the predictions meet such criterion.

**Figure 9:**
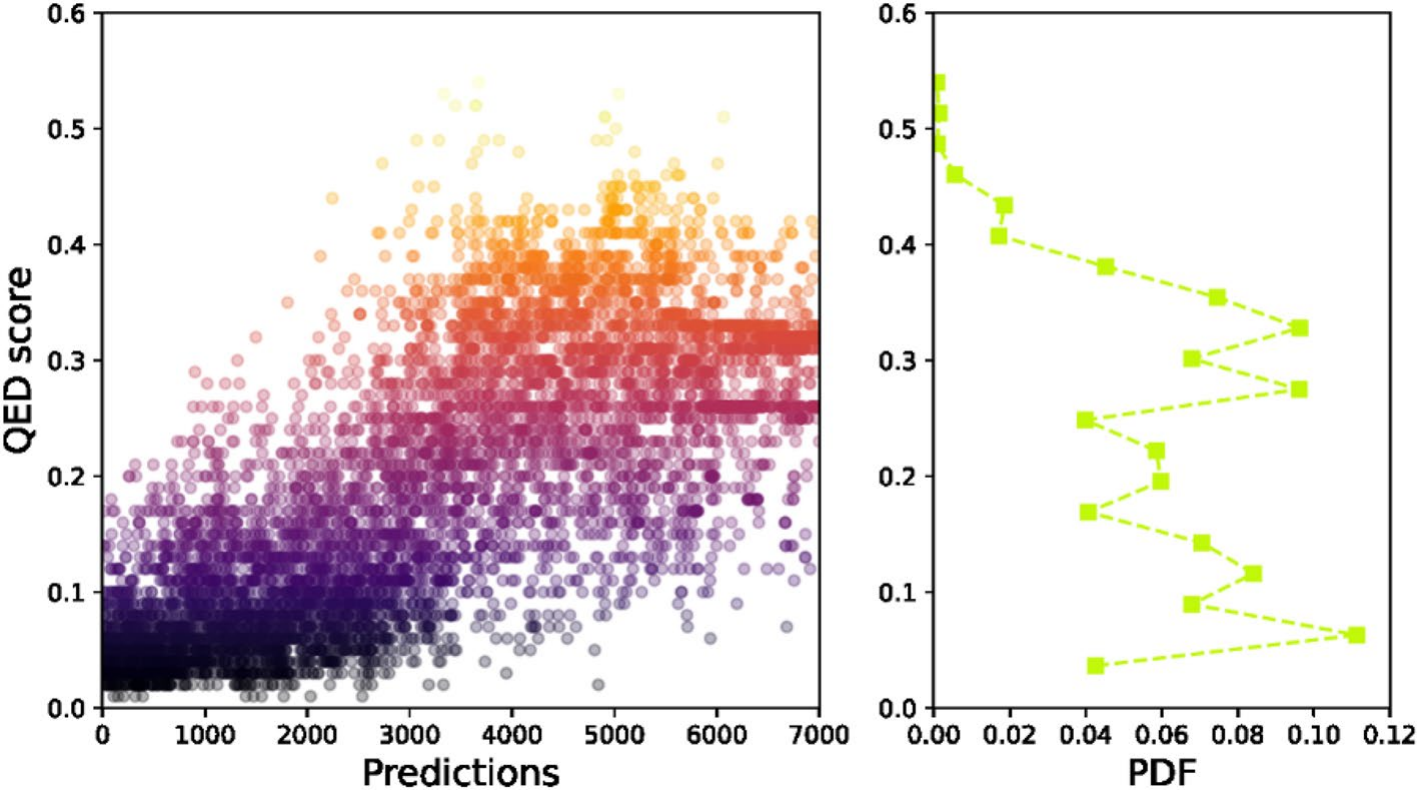
The quantitative estimate of drug-likeness (QED) score of the predicted ligand and the probability distribution (PDF).

The estimation of QED score of 7000 predictions and the corresponding probability distribution are indicated by Fig. 9. A clear increasing fashion could be recognized between the range from 0.0 to 0.6. Moreover, we can see from the probability distribution that the predictions span comparably between 0.05 and 0.35. The QED score for host molecule is 0.25, which means about half of the predictions would have higher QED score. Similarly, we chose 0.3 as the threshold for QED estimation to determine potential candidates from 7000 predictions. With such a value, 24.9% of the predictions are considered as the obtained.

Based on the above results, three thresholds have been defined, they are − 10.3 kcal mol^−1^ for binding energy, 0.72 for SA score and 0.3 for QED score. As a kind reminder, a successful prediction should meet all three characteristics at the same time. Therefore, according to those thresholds, we filtered out three candidates which have been further analyzed by molecular docking simulations for probing the biological activity. Meanwhile, the experimentally synthesized Cd complex has also been examined by molecular docking simulation for understanding the potential mechanism of biological activity.

### Validation of the predictions

**Scheme 1:**
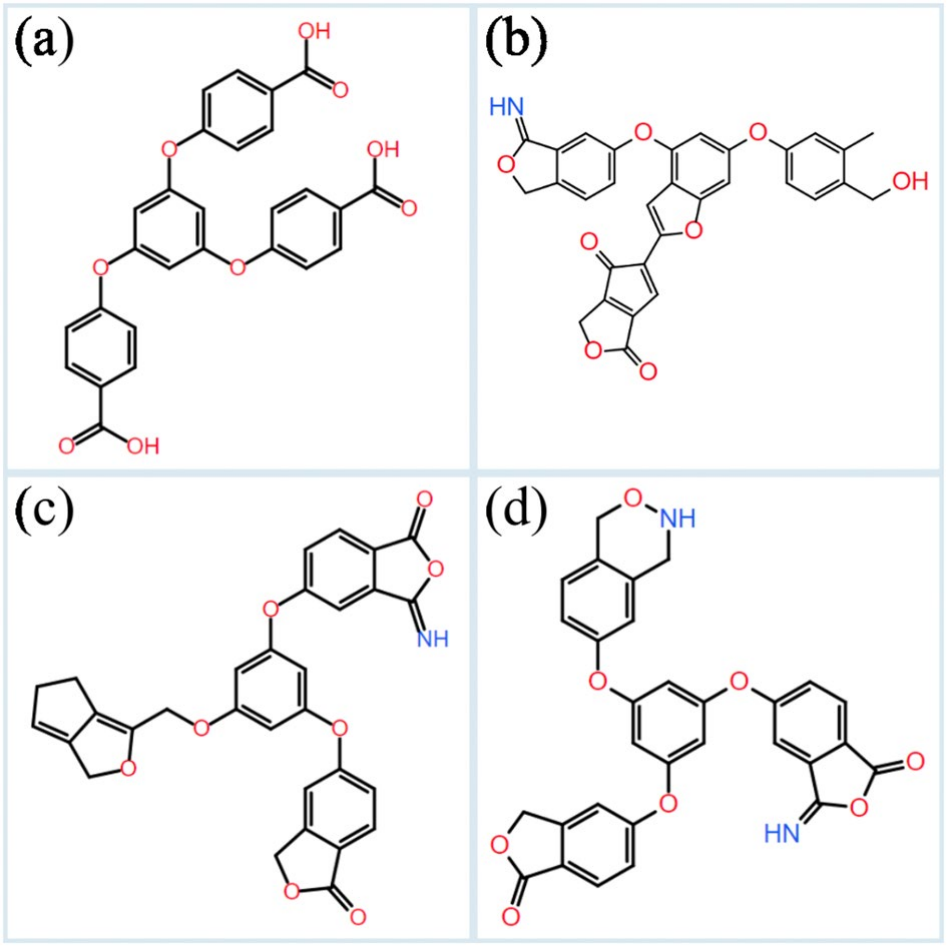
Schematical illustration of the ligand used in the Cd complex (**a**) and three outstanding ligand structures predicted through the generative automated model. Explicitly, the binding energy, SA and QED scores for ligand (**b**) are − 10.3 kcal mol^−1^, 0.72 and 0.31, the values for ligand (**c**) are − 10.5 kcal mol^−1^, 0.72 and 0.39, and the values for ligand (**d**) are − 10.7 kcal mol^−1^, 0.73 and 0.30, respectively.

Based on the three criterions of binding energy, SA and QED score, three predicted candidates are investigated by molecular docking simulations for validating the generative model and investigating their biological activity. The chemical sketches are shown in Scheme 1, where Scheme 1a is the host ligand from the Cd complex and Scheme 1b–d are the predicted molecules. It can be seen that the predicted structures still main their three-armed feature, although the origin carboxyl groups are replaced by different kinds of functional groups. As has been mentioned earlier, the binding energy, SA and QED scores for host molecule are − 8.5 kcal mol^−1^, 0.91 and 0.25. In contrast, these characteristics for predicted molecules are − 10.3, − 10.5 and − 10.7 kcal mol^−1^ (binding energy), 0.72, 0.72 and 0.73 for SA score, and 0.31, 0.39 and 0.30 for QED score, respectively.

**Figure 10:**
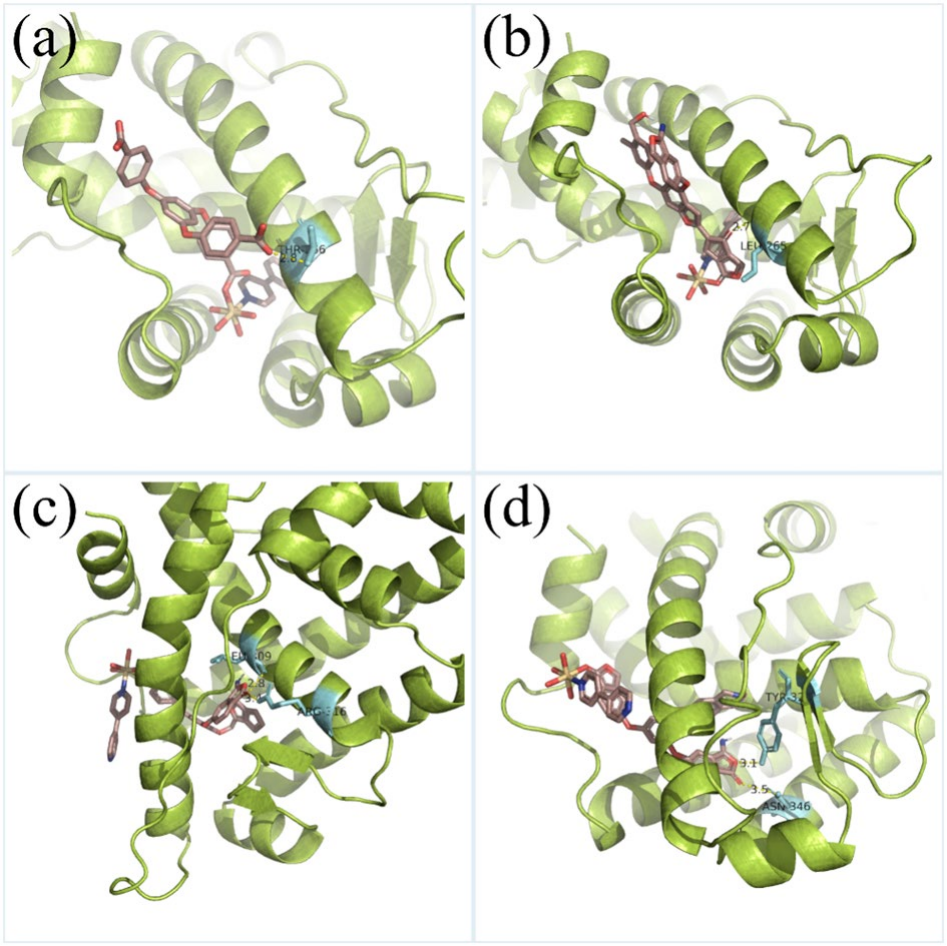
The binding poses for the Cd complexes with the ligands that are shown in Scheme 1, for which the binding interactions are studied in details from the atomistic point of view. The binding interactions as well as the active resides are shown explicitly.

Finally, the biological activities of host as well as the predicted molecules are investigated together with the Cd ion and the co-ligand. For each of the Cd complex, 10 possible binding poses are calculated, and the binding pose which presents the lowest binding energy has been further analyzed. The binding poses are shown in Fig. 10. The binding energy for the experimentally synthesized Cd complex (Fig. 10a) is − 8.72 kcal mol^−1^, and the inclusion constants is 406.65 nM. As expected, the binding interaction is formed by the carboxyl group, which interacts with residue THR-266 with a binding distance of 2.8 Å. The binding pose in Fig. 10b has a binding energy of − 7.51 kcal mol^−1^ and an inhibition constant of 3.15 μM. The carbonyl group interacts with residue LEU-265 with a polar distance of 2.7 Å. The binding pose in Fig. 10c has a binding energy of − 10.36 kcal mol^−1^ and an inhibition constant of 25.56 nM. Different from the from the first two binding poses, two binding interactions are observed, the carbonyl group interacts with residues LEU-309 and ARG-316, the corresponding distances are 2.8 and 3.4 Å. In Fig. 10d, the calculated binding energy and inhibition constant are − 12.15 kcal mol^−1^ and 1.25 nM. Two binding interactions are formed, one is from the carbonyl group and the other is from the ether group. The binding distances are 3.1 Å with residue TYR-326 and 3.5 Å with residue ASN-246. It can be concluded that the Cd complex with predicted structure as ligand has comparable (Fig. 10b) or better (Fig. 10c and d) anti-leukemia activity in comparison with the experimentally synthesized Cd complex. Therefore, the generative model based on the reinforcement learning has been validated as an effective tool for screening novel drug molecules using a known structure as the template.

## Conclusion

In summary, we have successfully constructed a complex [Cd_6_(L)_4_(bipy)_3_(H_2_O)_2_·H_2_O] (**1**) based on a tricarboxylic acid and bipy ligands in the same hydrothermal situation. The experiment proved that **1** shows an interesting 3D framework structure based on a trinuclear metal clusters, which has been identified via XRD, elemental analysis and IR.

Based on chemical synthesis, we successfully prepared HA/CMCS hydrogel, inheriting not only excellent biocompatibility from natural polysaccharides but also presenting a unique 3D porous network structure. The evenly distributed and highly concentrated internal pores provide an ideal environment for loading and releasing drug molecules, demonstrating great potential as an outstanding drug carrier. Using Dox as a model drug, we designed and synthesized structurally novel Dox-loaded metal gel particles. To further verify the effective encapsulation of Dox-loaded MOF by the hydrogel, we conducted fluorescence tests to examine its fluorescence effect and release time, followed by component analysis through TGA. Subsequently, we treated leukemia cells with this system at different concentrations. The system significantly upregulated the expression of the pro-apoptotic gene Bax, inducing apoptosis in leukemia cells. This system holds promise for development as a novel therapy for leukemia.

Through molecular docking simulations, the generative model was shown to predict new constructions featuring low binding energies, high SA and QED scores, and the predicted structures have comparable or even better biological activity compared to experimentally synthesized drug molecules. The generative model based on the reinforcement learning can be used an effective tool to design novel drug molecules when use a known structure as the template.

## Data Availability

The data used to support the findings of this study are included within the article.
